# Down-regulation of HSP60 Suppresses the Proliferation of Glioblastoma Cells via the ROS/AMPK/mTOR Pathway

**DOI:** 10.1038/srep28388

**Published:** 2016-06-21

**Authors:** Haiping Tang, Jin Li, Xiaohui Liu, Guihuai Wang, Minkui Luo, Haiteng Deng

**Affiliations:** 1 MOE Key Laboratory of Bioinformatics, School of Life Sciences, Tsinghua University, Beijing, 100084, China; 2 Department of Neurosurgery, Changgung Hospital Affiliated to Tsinghua University, Beijing, 100084, China; 3 Molecular Pharmacology and Chemistry Program, Memorial Sloan-Kettering Cancer Center, New York, 10065, United States

## Abstract

Glioblastoma is a fatal and incurable cancer with the hyper-activated mTOR pathway. HSP60, a major chaperone for maintenance of mitochondrial proteostasis, is highly expressed in glioblastoma patients. To understand the effects of HSP60 on glioblastoma tumorigenesis and progression, we characterized the HSP60-knockdowned glioblastoma cells and revealed that HSP60 silencing markedly suppressed cell proliferation and promoted cell to undergo the epithelial-mesenchymal transition (EMT). Proteomic analysis showed that ribosomal proteins were significantly downregulated whereas EMT-associated proteins were up-regulated in HSP60-knockdowned U87 cells as confirmed by a distinct enrichment pattern in newly synthesized proteins with azido-homoalanine labeling. Biochemical analysis revealed that HSP60 knockdown increased reactive oxygen species (ROS) production that led to AMPK activation, similarly to the complex I inhibitor rotenone-induced AMPK activation. Activated AMPK suppressed mTORC1 mediated S6K and 4EBP1 phosphorylation to decrease protein translation, which slowed down cell growth and proliferation. On the other hand, high levels of ROS in HSP60 knockdowned or rotenone-treated U87 cells contributed to EMT. These results indicate that HSP60 silencing deactivates the mTOR pathway to suppress glioblastoma progression, suggesting that HSP60 is a potential therapeutic target for glioblastoma treatment.

Glioblastoma (GBM) is the most common and lethal brain tumor in adults^1^. GBM is classified by the World Health Organization as the grade IV gliomas. Complete surgical removal of GBM tumors is difficult due to its invasion to the surrounding brain tissues^2^. GBM patients are not well responded to radiotherapy and chemotherapy^3^,^4^, which leads to an extremely poor prognosis in GBM patients^5^. The median survival of GBM patients is less than one year after diagnosis^6^, and the five year survival rates of GBM patients are less than 10%^7^. Three dysregulated pathways were identified in GBM cells including receptor tyrosine kinase (RTK) signaling, retinoblastoma (RB) signaling and TP53 signaling^8^. As a result of the enhanced RTK signaling, mTORC1 signaling is hyperactivated in GBM that promotes growth and proliferation and can be a compelling therapeutic target in GBM treatment^2^. However, rapamycin, the mTORC1 inhibitor, was failed in clinical trials due to insufficiently suppression of mTORC1 signaling, rapamycin resistance or activation of the downstream effectors^9^. Finding a new target to suppress mTORC1 signaling is, therefore, important for development of new therapeutic strategies for GBM. It is known that activated AMPK phosphorylates raptor to decrease mTORC1 activity^10^. However, AMPK mediated growth reduction in GBM has not yet been reported.

Heat shock protein 60 (HSP60) is one of the major chaperones in mitochondria for assisting protein folding, transportation and degradation to maintain mitochondrial proteostasis^11^,^12^,^13^,^14^. HSP60 plays tumor-type dependent roles with pro-apoptotic or pro-survival functions in tumorigenesis and progression^15^. Samali *et al.*^16^ reported that HSP60 interacted with pro-caspase3 that accelerated its activation *in vitro* and *in vivo*. However, Kirchhoff *et al.*^17^ showed that cytosolic HSP60 played an anti-apoptotic role in cardiac myocytes through interactions with pro-apoptotic Bax and Bak proteins that were also considered as an important factor in preventing apoptosis in tumor cells. As the result, HSP60 was down-regulated in bronchial adenocarcinoma, vesical transitional cell carcinoma and carcinosarcoma^18^,^19^,^20^,^21^ whereas the high HSP60 expression was presented in cervical cancer, prostate cancer, breast cancer and GBM^22^,^23^,^24^,^25^.

Effects of HSP60 on GBM progression has not been well examined. In the present work, we established multiple stable cell lines, in which HSP60 expression was knocked down. We carried out both quantitative proteomics and AHA labeling experiments to understand the mechanisms underlying HSP60 mediated cellular processes. AHA, an analog of methionine with azide group, is recognized by methionine tRNA and incorporated into newly synthesized proteins. These newly synthesized proteins can be enriched by click chemistry and subsequently identified by LC-MS/MS analysis. AHA labeling was a method of choice for studying regulation of protein synthesis. Our results revealed that HSP60 silencing in U87 cells enhanced cellular ROS production through disruption of respiratory complex I and markedly suppressed cell growth and proliferation through activating the ROS-AMPK-mTOR pathway, suggesting that HSP60 is a potential target for GBM treatment. We also demonstrated that bioorthogonal noncanonical amino acid tagging is a useful tool for examining cellular proteostasis network.

## Results

### HSP60 knockdown suppresses cell growth and proliferation and increases susceptibility to oxidative stress

**Figure 1:**
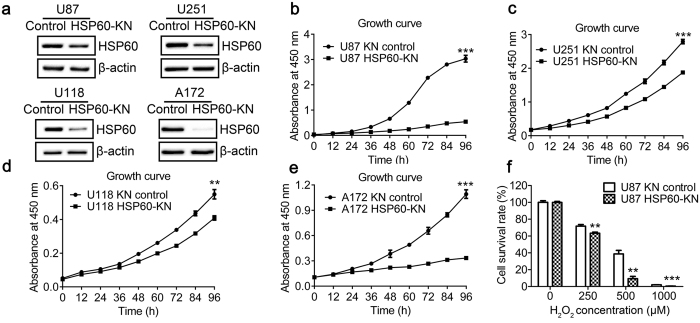
HSP60 knockdown in glioblastoma cells suppressed cell growth. (**a**) Western blotting images showed that the expression levels of HSP60 were decreased in HSP60-KN cells compared to control cells. (**b**) Growth curve of HSP60-KN-U87 and the control cells. (**c**) Growth curve of HSP60-KN-U251 and the control cells. (**d**) Growth curve of HSP60-KN-U118 and the control cells. (**e**) Growth curve of HSP60-KN-A172 and the control cells. (**f**) The cell survival rate of HSP60-KN-U87 and their control cells treated with different concentration of H_2_O_2_. Data were analyzed using student’s t test. *p < 0.05, **p < 0.01 and ***p <  0.001. *p < 0.05 is considered statistically significant. Error bars represent ± SEM.

To explore the effects of HSP60 expression on GBM cell proliferation, we constructed stable cell lines in which HSP60 expression was knocked down by small hairpin RNA interference. The cells transfected with a scrambled shRNA with no homology in human genome was used as the control. HSP60 silencing efficacy in U87, U251, U118 and A172 cells was examined by qPCR and western blotting (Fig. 1(a) and Supplementary Fig. S1), showing that the expression levels of HSP60 were decreased by approximately 50% in HSP60-knockdown cells. HSP60 knockdown significantly decreased the cell growth rate in all four cell lines (Fig. 1(b–e)), but did not decrease the growth in other cell lines such as 293T, A549, A2780 and 786-O cells (data not shown). The results indicate that HSP60 knockdown only suppresses cell proliferation in GBM cells.

Next, we examined whether HSP60 knockdown cells were more sensitive to oxidative stress. The cell viability of H_2_O_2_-treated cells was determined by CCK-8 assay (Fig. 1(f)). When cells were treated with 500 μM H_2_O_2_ for 12 h, cell survival rates were 40% and 10% for the control and HSP60-KN-U87 cells, respectively. A significant decrease in cell survival confirmed that HSP60-KN-U87 cells are more susceptible to oxidative stress.

### Identification of the differentially expressed proteins between the control and HSP60-KN cells

**Figure 2:**
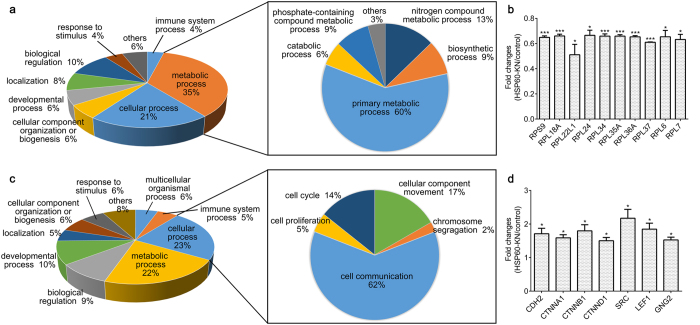
Proteomic analysis of differentially expressed proteins between HSP60-KN-U87 and control cells. (**a**) GO analysis of the down-regulated proteins in HSP60-KN-U87 cells compared to the control cells. (**b**) Graphical representation of TMT ratios for subunits of ribosome in HSP60-KN-U87 cells compared to the control cells. (**c**) GO analysis of the up-regulated proteins in HSP60-KN-U87 cells compared to the control cells. (**d**) Expression levels of proteins in Wnt/β-catenin pathway were up-regulated in HSP60-KN-U87 cells compared to the control cells. Data were analyzed using student’s t test. *p < 0.05, **p < 0.01 and ***p < 0.001. *p < 0.05 is considered statistically significant. Error bars represent ± SEM.

Quantitative proteomics analysis of the control and HSP60-KN-U87 cells identified 6877 proteins in biological triplicates. Based on the average reporter ion ratios from TMT analysis, 100 proteins were significantly up-regulated (ratio > 1.5, p value < 0.05) and 170 proteins were significantly down-regulated (ratio < 0.67, p value < 0.05) in HSP60-KN-U87 cells compared to control cells (Supplementary Tables S1 and S2). The Gene Ontology (GO) was used to analyze the biological relevance of the differentially expressed proteins with Panther (www.pantherdb.org/). As shown in Fig. 2, the majority of the differentially expressed proteins participated in the cellular process and the metabolic process. Among the down-regulated proteins, 116 proteins are associated with the primary metabolic process, which are the dominant difference between HSP60-KN-U87 and the control cells (Fig. 2(a)). We also found that ten ribosomal proteins were down-regulated in HSP60-KN-U87 cells (Fig. 2(b) and Table 1), suggesting that the protein synthesis rate in HSP60-KN cells was decreased. Among the up-regulated proteins, 26 proteins are associated with cell communication (Fig. 2(c)), in which seven proteins associated with the WNT/Catenin pathway were up-regulated in HSP60-KN cells (Fig. 2(d) and Table 1). HSP60 knockdown induced changes in the expression levels of RPS9, RPL18A, CDH2 (N-cadherin), CTNNB1 (β-catenin), SRC and LEF1 were further confirmed by western blot analysis (Supplementary Fig. S2).

*HSP60-silencing or rotenone treatment induces ROS overproduction in U87 cells.* 

**Figure 3:**
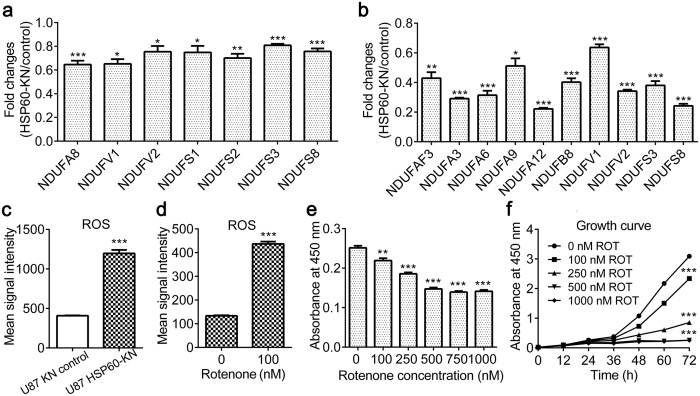
HSP60 knockdown disrupts complex I integrity leading to ROS overproduction. (**a**) Graphical representation of TMT ratios for subunits of respiratory complex I in HSP60-KN-U87 cells compared to the control cells. (**b**) Graphical representation of TMT ratios for newly synthesized subunits of respiratory complex I in HSP60-KN-U87 cells compared to the control cells. (**c**) Graphical representation of ROS levels of HSP60-KN-U87 cells compared to the control cells. (**d**) Graphical representation of ROS levels of U87 cells treated with 100 nM rotenone for 1 h. (**e**) Growth of U87 cells was suppressed when treated with different concentrations of rotenone for 24 h. (**f**) Growth curve of U87 cells treated with different concentrations of rotenone. Data were analyzed using student’s t test. *p < 0.05, **p < 0.01 and ***p < 0.001. *p < 0.05 is considered statistically significant. Error bars represent ± SEM.

**Figure 4:**
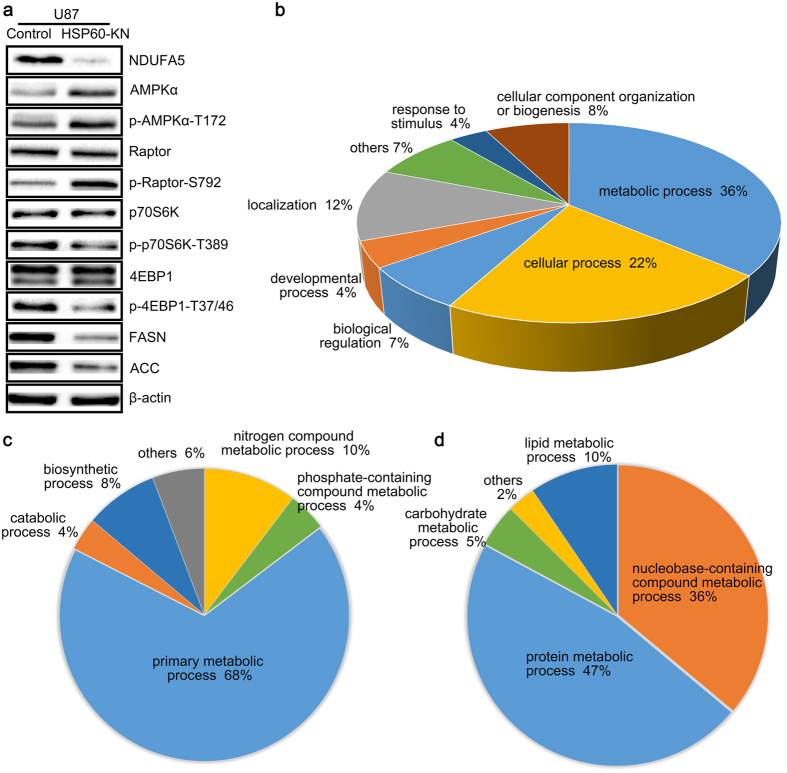
HSP60-silencing suppressed protein synthesis through the ROS/AMPK/mTOR pathway. (**a**) Western blotting images of NDUFA5, AMPK, raptor, P70S6K, 4EBP1, FASN and ACC in HSP60-KN cells compared to the control cells. (**b–d**) GO analysis of the decreased newly synthetic proteins in HSP60-KN cells compared to the control cells showing that proteins in protein metabolic process and nucleobase-containing compound metabolic process exhibited a lower synthesis rate in HSP60-KN-U87 cells.

Proteomics showed that seven subunits of respiratory complex I were downregulated in three biological replicate analysis of the control and HSP60-KN-U87 cells (Fig. 3(a) and Table 2) as confirmed by western blotting of NADH dehydrogenase ubiquinone 1 alpha subcomplex subunit 5 (NDUFA5), NADH dehydrogenase ubiquinone flavoprotein 1 (NDUFV1), NADH dehydrogenase ubiquinone flavoprotein 2 (NDUFV2) and NADH dehydrogenase ubiquinone iron-sulfur protein 3 (NDUFS3) in HSP60-KN-U87 cells (Fig. 4(a) and Supplementary Fig. S2), indicating that the integrity of the respiratory complexes in HSP60-KN-U87 cells was compromised. To clarify the underlying mechanism responsible for the downregulation of respiratory complex I, we carried out AHA experiment to enrich newly synthesized proteins. By quantitative proteomics, we identified that the synthesis rate of all 10 subunits of respiratory complex I was greatly decreased in HSP60-KN-U87 cells (Fig. 3(b) and Supplementary Table S3). This demonstrates that HSP60 silencing disrupts the mitochondrial proteostasis via slowing down protein synthesis rate.

It has been known that the cellular ROS was mainly produced from the respiratory complex I and respiratory complex III^26^. We, therefore, tested whether HSP60 knockdown caused ROS overproduction. The cellular ROS level was measured with the CellROX^®^ Deep Red kit and showed that HSP60 knockdown increased ROS production by about 3 fold, indicating that both mitochondrial proteostasis and ROS homeostasis were disrupted in HSP60-KN-U87 cells (Fig. 3(c)). To further confirm that HSP60-silencing induced downregulation of respiratory complex I was responsible for ROS overproduction, we treated U87 cells with 100 nM rotenone for 1 h, an inhibitor of respiratory complex I, which also resulted in ROS overproduction (Fig. 3(d)). Furthermore, we demonstrated that rotenone treatment also slowed down the growth and proliferation of U87 cells (Fig. 3(e,f)), similarly as those observed for HSP60-KN-U87 cells.

### HSP60-silencing suppressed cell growth through the ROS/AMPK/mTOR pathway

Western blotting was employed to investigate the ROS-downstream signaling pathways underlying HSP60 silencing mediated suppression of cell proliferation. Phosphorylation of AMPKα at T172 were significantly increased in HSP60-KN-U87 cells (Fig. 4(a) and Supplementary Fig. S3), indicating that ROS activated AMPK pathway. Targets of activated AMPK pathway include the mTOR pathway and fatty acid synthesis, in which AMPK directly phosphorylates raptor to prevent the formation of mTORC1^27^. Indeed, western blotting showed that the phosphorylation of raptor was significantly increased (Fig. 4(a) and Supplementary Fig. S3), which consequently resulted in significantly decreased S6K and 4EBP1 phosphorylation (Fig. 4(a) and Supplementary Fig. S3), suggesting that protein translation was inhibited in HSP60-KN cells through the ROS/AMPK/mTOR pathway.

These results were further validated by the AHA labeling. Using this approach, we identified 3704 newly synthesized proteins, in which the levels of 706 proteins were significantly decreased (ratio < 0.67, p value < 0.05) and 143 proteins were significantly increased (ratio > 1.5, p value < 0.05) in HSP60-KN-U87 cells compared to the control cells (Supplementary Table S3 and S4). The difference in numbers of proteins with altered synthesis rates between these two cell lines indicates that HSP60 knockdown slows down protein translation. This was further validated by the western blot analysis of AHA labeled proteins, showing that HSP60 knockdown inhibited the protein translation (Supplementary Fig. S4). GO analysis showed that majority of the decreased synthetic proteins were associated to the metabolic process and cellular process (Fig. 4(b,c)). Synthesis rates for proteins associated with protein metabolism were lower in HSP60-KN-U87 cells (Fig. 4(d)). Especially, levels of 91 newly synthesized proteins related to ribosomes were lower in HSP60-KN-U87 cells than those in the control cells (Supplementary Table S3), in consistent with the proteomic results. On the other hand, levels of newly synthesized proteins associated with cell communication were higher in HSP60-KN-U87 cells than those in the control cells (Supplementary Table S4). Thus, HSP60 knockdown induced a distinct enrichment pattern in newly synthesized proteins, demonstrating that proteostasis state was regulated by protein synthesis and HSP60 functions as a switch for tuning expressions of proteins of anabolic process and those associated with cell communication.

**Figure 5:**
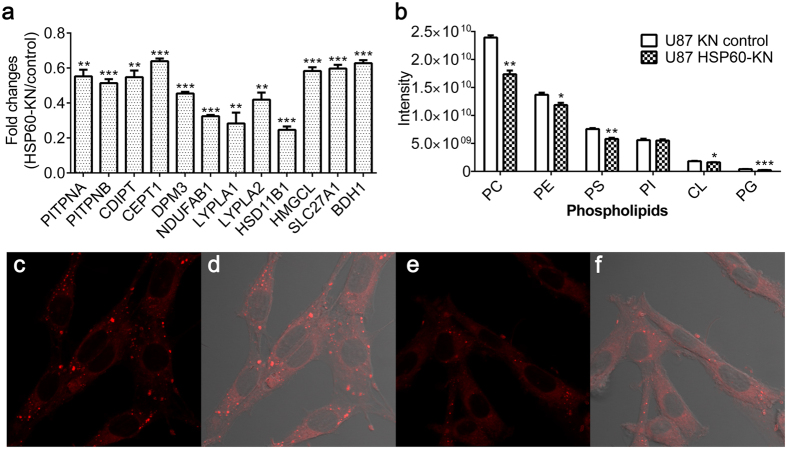
Lipid synthesis was decreased in HSP60-KN-U87 cells compared to the control cells. (**a**) Expression levels of proteins in lipid metabolism were down-regulated in HSP60-KN-U87 cells compared to the control cells. (**b**) Lipidomics analysis showed that five classes of phospholipids were decreased in HSP60-KN-U87 cells compared to the control cells. Data were analyzed using student’s t test. *p < 0.05, **p < 0.01 and ***p < 0.001. *p < 0.05 is considered statistically significant. Error bars represent ± SEM. (**c**,**d**) Nile red staining of the control cells with or without bright-field alignment. (**e**,**f**) Nile red staining of HSP60-KN cells with or without bright-field alignment.

Furthermore, levels of 12 newly synthesized proteins related to lipid metabolism were significantly lower in HSP60-KN-U87 cells than those in the control cells (Fig. 5(a) and Supplementary Fig. S5), suggesting that HSP60 knockdown mediated activation of AMPK pathway affected lipid metabolism. We conducted the lipidomic analysis and showed that five classes of phospholipids were decreased in HSP60-KN-U87 cells (Fig. 5(b)). Nile red staining also indicated that phospholipids in HSP60-KN-U87 cells were reduced (Fig. 5(c–f)), in consistent with the proteomics and western blot data showing that fatty acid synthase and acetyl-CoA carboxylase were down-regulated in HSP60-KN-U87 cells (Fig. 4(a)). All these results declared that HSP60-silencing suppressed cell proliferation and growth through ROS/AMPK axis.

### HSP60-silencing promoted cell to undergo EMT

**Figure 6:**
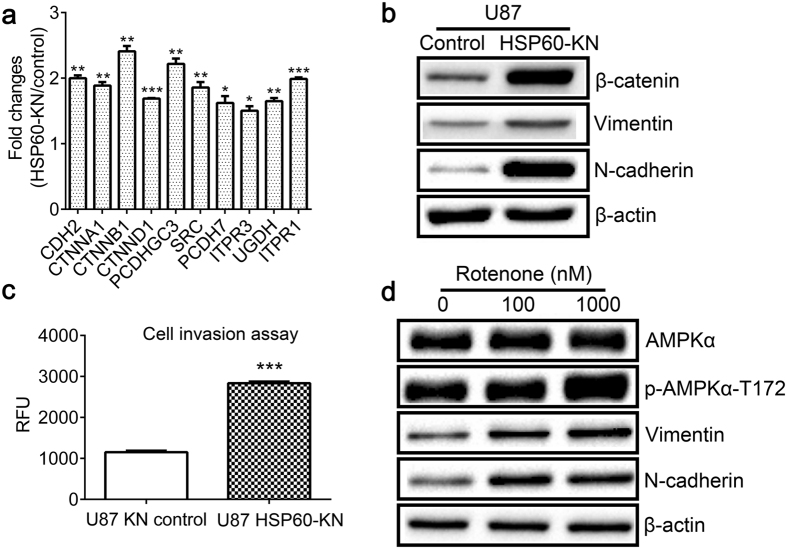
HSP60-silencing promoted cells to undergo the EMT process. (**a**) Graphical representation of TMT ratios for newly synthesized proteins in Wnt/β-catenin pathway that were up-regulated in HSP60-KN-U87 cells. (**b**) Western blotting images of β-catenin, vimentin, N-cadherin in the control cells and HSP60-KN cells. (**c**) Cell invasion assay showed that HSP60-KN cells displayed a higher invasiveness than control cells did. Data were analyzed using student’s t test. *p < 0.05, **p < 0.01 and ***p < 0.001. Error bars represent ± SEM. (**d**) Western blotting images of AMPK, vimentin, N-cadherin of untreated- and rotenone-treated U87 cells for 24 h.

AHA labeling results determined that levels of newly synthesized proteins in cell communication were higher in HSP60-KN-U87 cells than those in the control cells. For examples, HSP60 knockdown induced upregulation of the newly synthesized proteins associated with integrin pathway including Laminin subunit gamma-1, Focal adhesion kinase 1, Proto-oncogene tyrosine-protein kinase Src (SRC), Dedicator of cytokinesis protein 1, Integrin alpha-V, Paxillin, Engulfment and cell motility protein 2, Filamin-B, Integrin alpha-6, Alpha-actinin-1 and Collagen alpha-1(I) chain. Similarly, the WNT pathway associated proteins N-cadherin (CDH2), Catenin alpha-1(CTNNA1), β-catenin (CTNNB1), Catenin delta-1 (CTNND1), Proto-cadherin gamma-C3 (PCDHGC3), Protocadherin-7 (PCDH7), Inositol 1,4,5-trisphosphate receptor type 3 (ITPR3), UDP-glucose 6-dehydrogenase (UGDH) and Inositol 1,4,5-trisphosphate receptor type 1 (ITPR1) were increasingly synthesized in HSP60-KN-U87 cells compared to the control cells (Fig. 6(a) and Supplementary Table S4). HSP60 knockdown mediated activation of integrin and WNT pathway proposes that cells undergo EMT process as confirmed by the western blotting, showing that EMT markers N-cadherin and vimentin were significantly increased (Fig. 6(b) and Supplementary Fig. S6). Western blot analysis also revealed that both the cytoplasmic and nuclear levels of β-catenin were higher in HSP60-KN-U87 cells (Fig. 6(b) and Supplementary Fig. S7). It has been reported that nuclear accumulation of β-catenin initiated EMT in pancreatic cancer^28^. Consistent with these results, our data suggested that β-catenin played an important role in HSP60 silencing mediated EMT process. Moreover, cell invasion assay showed that HSP60-KN-U87 cells displayed a higher invasiveness than control cells did (Fig. 6(c)).

To clarify whether HSP60 knockdown mediated ROS/AMPK activation causes EMT, U87 cells were treated with rotenone and expression changes in selected proteins were probed by western blotting. As we expected, rotenone also induced activation of AMPK pathway and upregulation of EMT markers (Fig. 6(d) and Supplementary Fig. S6), which confirmed that ROS-induced AMPK activation was the underlying mechanism for EMT process in GBM cells.

## Discussion

GBM is the most common brain tumor with high relapse and mortality rate^29^. The current therapy is based on surgical excision followed by temozolomide chemotherapy and/or radiation^30^,^31^. New therapeutic approaches are urgently needed. Consistent with previous results that the high HSP60 expression was detected in GBM patients, we confirmed that HSP60-silencing suppressed the growth and proliferation in multiple GBM cell lines^32^. The high cellular ROS level and mesenchymal phenotype were also presented in HSP60-knockdown cells. Proteomic analysis and AHA labeling shed light on the mechanisms underlying HSP60-silencing enhanced ROS production, in which multiple subunits of the respiratory complex I were down-regulated in HSP60-KN-U87 cells. The respiratory complex I contains 45 subunits^33^, which must assemble together to form a mature holoenzyme for dehydrogenating NADH and transferring electrons to coenzyme Q. As a major site for ROS production^34^, early studies have shown that the down-regulation of complex I subunits causes dysfunction of the respiratory chain to generate excessive ROS^35^,^36^, which supports our finding that the high level of ROS is present in HSP60-KN-U87 cells. We further showed that AMPK was activated in HSP60-KN cells. Similar results were observed when cells were treated with rotenone. These results demonstrate that both HSP60 knockdown and rotenone-treatment activates ROS/AMPK pathway.

It has been known that AMPK regulates several cellular processes, including protein synthesis, fatty acid metabolism and glucose uptake^37^,^38^,^39^,^40^. Two different mechanisms were proposed for AMPK mediated inhibition of mTORC1^27^. AMPK phosphorylates TSC2 to inactivate Rheb^41^,^42^, an essential activator of mTORC1^43^; or AMPK directly phosphorylates raptor to prevent the formation of mTORC1 complex^44^. Our data showed that raptor phosphorylation was increased in HSP60-KN cells, which caused deactivation of the mTOR signaling. Consequently, targets of mTORC1 complex, S6K and 4EBP1 phosphorylation were decreased^45^,^46^, which ultimately led to 4EBP1 binding to eIF4E and prevented protein synthesis^47^,^48^. It was confirmed by the fact that the quantity of the newly synthesized proteins in HSP60-KN-U87 cells was significantly less than that synthesized in the control cells, especially for the ribosomal proteins and the complex I subunits.

Activated AMPK also regulated lipid metabolism including fatty acid synthesis and β-oxidation^37^. It has been known that AMPK phosphorylates acetyl CoA carboxylase 1, a rate limiting enzymes in fatty acid synthesis, to down-regulate fatty acid synthesis^49^ while AMPK also inhibits the activity of SREBP1, the transcription factor for ACC1 and FASN, to suppress the expression of ACC1 and FASN^50^. Consistent with those findings, we found that ACC and FASN were down-regulated in HSP60-KN cells, resulting in a markedly decrease in lipid synthesis, especially for phospholipids. We further demonstrated that HSP60-knockdowned or rotenone treated cells underwent EMT with enhanced cell motilities.

## Conclusions

In summary, our results provide mechanistic information for understanding functions of HSP60 in tumorigenesis and progression of GBM cells. We demonstrate that HSP60 played a key role in maintenance of mitochondrial proteostasis and ROS homeostasis. HSP60 silencing or rotenone treatment disrupted the integrity of the respiratory chain, leading to ROS overproduction and activation of AMPK pathway, which inhibited protein synthesis and drove cell to undergo EMT. Our data propose that HSP60 silencing attenuates the mTOR pathway to suppress cell proliferation and HSP60 is a potential therapeutic target for GBM treatment.

## Methods

### Cell Lines

Human glioblastoma cell line U87, U251, U118, A172 and human embryonic kidney 293T were obtained from cell bank of Chinese Academy of Sciences (Shanghai, China). U87 cells, U251 cells, U118 cells, A172 cells and 293T cells were grown in DMEM media (Wisent, Montreal, QC) supplemented with 10% fetal bovine serum (Wisent, Montreal, QC) and 1% penicillin/streptomycin (Wisent, Montreal, QC) at 37 °C in a humidified incubator with 5% CO_2_.

### HSP60 knock down stable transfection cell line

The shRNA sequence used for targeting HSP60 was based on previous report^51^ and further confirmed by NCBI BLAST. A scrambled shRNA with no homologous sequence been found in the human genome was used as negative control. The shRNA sequences were shown in Supplementary Table S5. The shRNAs were cloned into plasmid pLL3.7 and co-transfected with pMD2.G, pMDLg/pRRE and pREV-Rev into 293T cells using Lipofectamine 2000 (Invitrogen, NY) to package lentiviral particles. After transfection for 48 h, Supernatants containing lentiviral particles were harvested and used to infect U87, U251, U118 and A172 cells in the presence of 6 μg/ml of polybrene for 5 h.Cells were cultured in fresh medium for 96 h after infections and sorted by flow cytometer with GFP positive to generate stable cell lines.

### Cell proliferation assay with CCK-8

Cells were seeded in 96-well plates (2,000 cells per well). Cell proliferation rate was measured with the Cell Counting Kit-8 (CCK-8) according to the manufacturer’s instructions (Dojindo Laboratories, Japan). Briefly, CCK-8 reagents were added into wells after cells grew for 0, 12, 24, 36, 48, 72, 84, 96 h respectively or treated with rotenone for 0, 12, 24, 36, 48, 60, and 72 h. The plates were incubated at 37 °C for 2 h. Absorbance at 450 nm was measured after incubation.

Cell viability assay of HSP60 knockdown cells treated with hydrogen peroxide. 

Effects of hydrogen peroxide on control and HSP60 knockdown cells were analyzed with the CCK-8 kit. Briefly, Cells were treated with hydrogen peroxide (0, 250, 500 and 1,000 μM) for 12 h. The CCK-8 reagent was added to treated cells and incubated at 37 °C for 2 h. Optical density (OD) was measured at 450 nm with a microplate reader.

### Proteomic analysis of newly synthesized proteins

5 × 10^6^ cells were seeded onto 100 mm dishes supplemented with 10 ml DMEM medium and incubated in CO_2_ incubator overnight. Cells were washed twice with PBS, and supplemented with DMEM medium with 40 μM L-methionine or L-Azidohomoalanine. Cells were then incubated in CO_2_ incubator for 4 h. Cells were harvested and washed twice with PBS and lysed in PBS buffer containing 0.1% SDS, 1% NP-40 and protease inhibitor cocktail. After sonication, cell lysates were centrifuged at 14,000 × g for 20 min at 4 °C. Proteins were quantified with BCA kit. 500 μg proteins were reacted with biotin alkyne (Thermo, Waltham, MA) supplemented with 1 mM CuSO_4_ and 5 mM vitamin C for 1 h at room temperature. After methanol precipitation, the precipitates were redissolved in RIPA buffer and incubated with 40 μl streptavidin sepharose overnight at 4 °C. The beads were washed three times with PBS buffer containing 1% NP-40, then washed three times with PBS buffer. Finally, the newly synthesized proteins were eluted with 2× loading buffer and loaded onto SDS-PAGE gel. The gel bands were excised and digested with the standard in-gel digestion protocol. The digested peptides were extracted twice with 50% acetonitrile aqueous solution containing 0.1% formic acid for 60 min. Extracts were then dried in a Speed Vac and labelled with the TMT reagent (Thermo, Waltham, MA) according to the manufacturer’s protocol. Labelled peptides were mixed together and analyzed by LC-MS/MS. The peak lists from LC-MS/MS analysis were generated with the SEQUEST^TM^ searching algorithm in the Proteome Discoverer software (version 1.4.1.14). The generated MS/MS spectra were searched against the Uniprot Human database (release date of November 20, 2015; 20193 entries) using the SEQUEST HT searching engine. The search criteria were as follows: full tryptic specificity was required; two missed cleavage was allowed; carbamidomethylation (C) and TMT sixplex (K and N-terminal) were set as the fixed modifications; the oxidation (M) was set as the variable modification; precursor ion mass tolerances were set at 10 ppm for all MS acquired in an orbitrap mass analyzer; and the fragment ion mass tolerance was set at 0.02 Da for all MS2 spectra acquired. The peptide false discovery rate was calculated using Percolator provided by Proteome Discoverer software. When the q value was smaller than 1%, the peptide spectrum match was considered to be correct. False discovery was determined based on peptide spectrum match when searched against the reverse, decoy database. Peptides only assigned to a given protein group were considered as unique. Relative protein quantification was performed using the Proteome Discoverer software (Version 1.4.1.14) according to manufacturer’s instructions on the six reporter ion intensities per peptide. Protein ratios were calculated as the median of all peptide hits belonging to a protein. Proteins with ratios smaller than 0.67 and p-value < 0.05 were considered as down-regulated proteins while proteins with ratios greater than 1.5 and p-value < 0.05 were considered as up-regulated proteins. Tryptic peptides from bovine serum albumin were used as the quality control (QC) sample to evaluate the performance of mass spectrometry system.

### Protein Separation by two dimensional HPLC and Proteomics Analysis

Proteomic analysis was carried out as previously described^52^. Briefly, Proteins of infected U87 cells were extracted with 8 M urea, and 100 μg of protein was reduced by adding 5 mM dithiotheitol for 1 h at room temperature and then alkylated by 12.5 mM iodoacetamide for 1 h at room temperature protected from light. The protein samples were finally digested with trypsin for 20 h at room temperature. The digested peptides were purified using Sep-Pak C18 cartridges and labeled with TMT reagents (Thermo, Waltham, MA) according to the manufacturer’s protocol. Labelled peptides were mixed together, desalted by Sep-Pak C18 cartridges and separated by reverse phase (RP) chromatography. The first dimension RP separation by microLC was performed on an Ultimate 3000 System (Thermo, Waltham, MA) by using a Xbridge C18 RP column (5 μm, 150 Å, 250 mm × 4.6 mm i.d., Waters). Mobile phases A (2% acetonitrile, adjusted pH to 10.0 using NH_3_·H_2_O) and B (98% acetonitrile, adjusted pH to 10.0 using NH_3_·H_2_O) were used to develop a gradient. The eluted peptides were monitored at 214 nm and collected every minute. The fractions were dried and reconstituted in 20 μL of 0.1% (v/v) FA in water for the second dimension LC-MS/MS analysis at a low pH value. The peptides were separated by a C18 column (75 μm inner-diameter, 150 mm length; Upchurch, Oak Harbor, USA) with a flow rate of 250 nL/min. Mobile phase A consisted of 0.1% formic acid, and mobile phase B consisted of 100% acetonitrile and 0.1% formic acid. The Orbitrap Q-Exactive mass spectrometer was operated in the data-dependent acquisition mode using Xcalibur 3.0 software. A full-scan followed by 20 data-dependent MS/MS scans were acquired using higher energy collisional dissociation with normalized collision energy of 35%. The data analysis was the same as described above. Proteomic analysis was carried out in biological triplicates.

### Detection of Cellular Reactive Oxygen Species (ROS)

The cellular ROS was measured using CellROX^®^ Deep Red Reagents (Invitrogen, Grand Island, NY) following manufacturer’s instructions. Briefly, cells were stained with 5 μM CellROX^®^ Deep Red Reagent and incubated at 37 °C for 30 min. Then, cells were washed with PBS and analyzed on a BD FACS Aria II Flow Cytometer (BD Biosciences, San Jose, CA).

### Western blotting

Cells were lysed in RIPA buffer supplemented with protease inhibitor cocktail and phosphatase inhibitor cocktail (Thermo, Waltham, MA). Equal amount of proteins were separated on the 12% SDS-PAGE gel and transferred onto a PVDF transfer membrane. Western blot analysis followed a standard procedure. P70 S6 Kinase, phospho-p70 S6 Kinase (Thr389) β-catenin and FASN antibodies were obtained from Sigma (St Louis, MO). AMPKα, phospho-AMPKα (Thr172), Raptor, phosphor-Raptor (Ser792), 4EBP1, phospho-4E-BP1 (Thr37/46), ACC, N-cadherin, SRC, LEF1 and β-actin were obtained from Cell Signaling Technology (Danvers, MA). NDUFV1, NDUFV2, NDUFS3, RPS9 and RPL18A were obtained from Proteintech (Chicago, IL). NDUFA5 antibody was obtained from Pierce (Rockford, IL). HSP60 antibody was obtained from Stressgen (Victoria, BC). Vimentin antibody was obtained from Proteintech (Chicago, IL). Histone H3 antibody was obtained from Beyotime(shanghai, China).

### Quantitative real-time PCR (qPCR)

Total RNA was isolated using the SV Total RNA Isolation System (Promega, Fitchburg, WI) according to the manufacturer’s instructions. cDNA were synthesized using the GoScript^TM^ Reverse Transcription System (Promega, Fitchburg, WI).All qPCRs were performed using the Roche LightCycler 96 System with SYBR green incorporation (Promega, Fitchburg, WI). The primers for all genes used in this study are from primerbank (https://pga.mgh.harvard.edu/primerbank/index.html) and listed in Supplementary Table S6. 18SrRNA was used as internal control. The relative mRNA level was calculated using 2^−ΔΔCt^ method.

### Lipid analysis

Lipids were extracted according to the method of Bligh and Dyer^53^. 5 × 10^6^ cells were suspended in 500 μl PBS, followed by addition of 3 ml of mixture of chloroform and methanol (V:V = 2:1). Samples were vortex for 30 s and centrifuged at 1,000 rpm for 5 min. The lower chloroform layer was collected and dried by nitrogen. Samples were reconstituted in chloroform and methanol solution (V:V = 2:1), separated with Cortecs C18 column (2.1 × 100 mm, Waters) and analyzed by an Q Exactive orbitrap mass spectrometer that was operated in both positive and negative mode using Xcalibur 3.0 software. A full-scan followed by 10 data-dependent MS/MS scans were acquired using higher energy collisional dissociation with stepped normalized collision energy of 15%, 30% and 45%. The data analysis was performed on LipidSearch software (Thermo, Waltham, MA). The search criteria were as follows: product search type was used, precursor ion mass tolerances were set at 8 ppm for all MS acquired in an orbitrap mass analyzer and the fragment ion mass tolerance was set at 15 ppm for all MS2 spectra acquired. Sample mixture was used as quality control to evaluate the performance of mass spectrometry.

### Nile red staining

Cells were fixed with 4% paraformaldehyde for 20 min and washed with PBS for three times. Then, cells were incubated with the Nile red solution (1 μg/ml fresh solution) for 20 min, washed with PBS for three times and imaged with co-focal microscopy (Zeiss, Jena, Germany).

### Invasion assay

Invasion assay was performed using QCM 24-well cell invasion assay kit (Millipore, Boston, MA) according to the manufacturer’s instructions. Briefly, cells were starved for 24 h prior to analysis. Cells were harvested and re-suspended in 1 ml Quenching Medium. 1.25 × 10^5^ cells were seeded in the insert containing serum free media while the serum containing media were added to the lower chamber. Cells then were incubated for 24 h in a CO_2_ incubator. Cells that invaded through the ECMatrix-coated membrane were lysed in Lysis Buffer/Dye Solution and incubated for 15 min at room temperature. RFU values were read with PerkinElmer’s EnSpire Multimode plate reader (Waltham, MA) in fluorescence mode using 480/520 nm filter set.

### Statistical analysis

Statistical analysis was carried out with GraphPad Prism 6.0 software by two sided unpaired *t* tests. P values of  < 0.05 were considered significant.

## Additional Information

**How to cite this article**: Tang, H. *et al.* Down-regulation of HSP60 Suppresses the Proliferation of Glioblastoma Cells via the ROS/AMPK/mTOR Pathway. *Sci. Rep.* **6**, 28388; doi: 10.1038/srep28388 (2016).

## Supplementary Material

Supplementary Tables

Supplementary Information

## Figures and Tables

**Table 1:** The list of the down-regulated ribosomal proteins and upregulated proteins associated with Wnt/β-catenin pathway in HSP60-KN-U87 cells.

Accession	Description	Score	Sequence coverage (%)	Ratio ± SD	p-value
P46781	40S ribosomal protein S9	340	66	0.65 ± 0.02	0
Q02543	60S ribosomal protein L18a	275	51	0.66 ± 0.02	0
Q6P5R6	60S ribosomal protein L22-like 1	29	25	0.51 ± 0.14	0.0269
P83731	60S ribosomal protein L24	184	48	0.67 ± 0.07	0.0131
P49207	60S ribosomal protein L34	129	44	0.66 ± 0.03	0.0001
P18077	60S ribosomal protein L35a	156	61	0.66 ± 0.02	0
P83881	60S ribosomal protein L36a	120	54	0.65 ± 0.02	0
P61927	60S ribosomal protein L37	33	45	0.61 ± 0	0
Q02878	60S ribosomal protein L6	304	31	0.65 ± 0.09	0.0196
P18124	60S ribosomal protein L7	459	49	0.63 ± 0.07	0.0108
P19022	Cadherin-2	52	15	1.71 ± 0.28	0.0485
P35221	Catenin alpha-1	574	61	1.59 ± 0.16	0.024
P35222	Catenin beta-1	379	60	1.8 ± 0.32	0.0487
O60716	Catenin delta-1	381	44	1.5 ± 0.17	0.0342
P12931	Proto-oncogene tyrosine-protein kinase Src	41	27	2.17 ± 0.46	0.0473
Q9UJU2	Lymphoid enhancer-binding factor 1	32	13	1.85 ± 0.31	0.0412
P59768	Guanine nucleotide-binding protein G(I)/G(S)/G(O) subunit gamma-2	30	52	1.53 ± 0.14	0.0235

**Table 2:** The list of the downregulated subunits of the respiratory complex I in HSP60-KN-U87 cells.

Accession	Description	Score	Sequence coverage (%)	Ratio ± SD	p-value
P51970	NADH dehydrogenase [ubiquinone] 1 alpha subcomplex subunit 8	24	12	0.65 ± 0.06	0.0004
P49821	NADH dehydrogenase [ubiquinone] flavoprotein 1, mitochondrial	62	49	0.65 ± 0.07	0.0113
P19404	NADH dehydrogenase [ubiquinone] flavoprotein 2, mitochondrial	23	23	0.75 ± 0.08	0.0353
P28331	NADH-ubiquinone oxidoreductase 75 kDa subunit, mitochondrial	80	30	0.75 ± 0.1	0.0439
O75306	NADH dehydrogenase [ubiquinone] iron-sulfur protein 2, mitochondrial	25	12	0.7 ± 0.06	0.0012
O75489	NADH dehydrogenase [ubiquinone] iron-sulfur protein 3, mitochondrial	25	13	0.81 ± 0.02	0.0002
O00217	NADH dehydrogenase [ubiquinone] iron-sulfur protein 8, mitochondrial	23	33	0.76 ± 0.04	0.0007

## References

[b1] Legler J. M. *et al.* Cancer surveillance series: brain and other central nervous system cancers: recent trends in incidence and mortality. J. Natl. Cancer Inst. 91, 1382–1390 (1999). 10451443 10.1093/jnci/91.16.1382

[b2] Cloughesy T. F., Cavenee W. K. & Mischel P. S. Glioblastoma: from molecular pathology to targeted treatment. Annu. Rev. Pathol. 9, 1–25 (2014). 23937436 10.1146/annurev-pathol-011110-130324

[b3] Dunn G. P. *et al.* Emerging insights into the molecular and cellular basis of glioblastoma. Gene Dev. 26, 756–784 (2012). 22508724 10.1101/gad.187922.112 PMC3337451

[b4] Masui K., Cloughesy T. F. & Mischel P. S. Molecular pathology in adult high-grade gliomas: from molecular diagnostics to target therapies. Neuropath. Appl. Neuro. 38, 271–291 (2012). 10.1111/j.1365-2990.2011.01238.x PMC4104813 22098029

[b5] Mrugala M. M. Advances and challenges in the treatment of glioblastoma: a clinician’s perspective. Discov. Med. 15, 221–230 (2013). 23636139

[b6] Stewart L. A. Chemotherapy in adult high-grade glioma: a systematic review and meta-analysis of individual patient data from 12 randomised trials. Lancet 359, 1011–1018 (2002). 11937180 10.1016/s0140-6736(02)08091-1

[b7] Stupp R. *et al.* Effects of radiotherapy with concomitant and adjuvant temozolomide versus radiotherapy alone on survival in glioblastoma in a randomised phase III study: 5-year analysis of the EORTC-NCIC trial. Lancet Oncol. 10, 459–466 (2009). 19269895 10.1016/S1470-2045(09)70025-7

[b8] Cancer Genome Atlas Research N. Comprehensive genomic characterization defines human glioblastoma genes and core pathways. Nature 455, 1061–1068 (2008). 18772890 10.1038/nature07385 PMC2671642

[b9] Cloughesy T. F. *et al.* Antitumor activity of rapamycin in a Phase I trial for patients with recurrent PTEN-deficient glioblastoma. PLos Med. 5, e8 (2008). 18215105 10.1371/journal.pmed.0050008 PMC2211560

[b10] Gwinn D. M. *et al.* AMPK phosphorylation of raptor mediates a metabolic checkpoint. Mol. Cell 30, 214–226 (2008). 18439900 10.1016/j.molcel.2008.03.003 PMC2674027

[b11] Lianos G. D. *et al.* The role of heat shock proteins in cancer. Cancer Lett. 360, 114–118 (2015). 25721081 10.1016/j.canlet.2015.02.026

[b12] Bukau B. & Horwich A. L. The Hsp70 and Hsp60 chaperone machines. Cell 92, 351–366 (1998). 9476895 10.1016/s0092-8674(00)80928-9

[b13] Fersht A. R. & Daggett V. Protein folding and unfolding at atomic resolution. Cell 108, 573–582 (2002). 11909527 10.1016/s0092-8674(02)00620-7

[b14] White G. W. N. *et al.* Simulation and experiment conspire to reveal cryptic intermediates and a slide from the nucleation-condensation to framework mechanism of folding. J. Mol. Biol. 350, 757–775 (2005). 15967458 10.1016/j.jmb.2005.05.005

[b15] Arya R., Mallik M. & Lakhotia S. C. Heat shock genes - integrating cell survival and death. J. Biosci. 32, 595–610 (2007). 17536179 10.1007/s12038-007-0059-3

[b16] Samali A., Cai J., Zhivotovsky B., Jones D. P. & Orrenius S. Presence of a pre-apoptotic complex of pro-caspase-3, Hsp60 and Hsp10 in the mitochondrial fraction of jurkat cells. EMBO J. 18, 2040–2048 (1999). 10205158 10.1093/emboj/18.8.2040 PMC1171288

[b17] Kirchhoff S. R., Gupta S. & Knowlton A. A. Cytosolic heat shock protein 60, apoptosis, and myocardial injury. Circulation 105, 2899–2904 (2002). 12070120 10.1161/01.cir.0000019403.35847.23

[b18] Cappello F., Di Stefano A., D’Anna S. E., Donner C. F. & Zummo G. Immunopositivity of heat shock protein 60 as a biomarker of bronchial carcinogenesis. Lancet Oncol. 6, 816–816 (2005). 16198989 10.1016/S1470-2045(05)70393-4

[b19] Cappello F. *et al.* HSP60 and HSP10 down-regulation predicts bronchial epithelial carcinogenesis in smokers with chronic obstructive pulmonary disease. Cancer 107, 2417–2424 (2006). 17048249 10.1002/cncr.22265

[b20] Lebret T. *et al.* Heat shock proteins HSP27, HSP60, HSP70, and HSP90: expression in bladder carcinoma. Cancer 98, 970–977 (2003). 12942564 10.1002/cncr.11594

[b21] Kamishima T. *et al.* Carcinosarcoma of the urinary bladder: expression of epithelial markers and different expression of heat shock proteins between epithelial and sarcomatous elements. Pathol. Int. 47, 166–173 (1997). 9088035 10.1111/j.1440-1827.1997.tb03735.x

[b22] Hwang Y. J. *et al.* Expression of Heat Shock Protein 60 kDa Is Upregulated in Cervical Cancer. Yonsei Med. J. 50, 399–406 (2009). 19568603 10.3349/ymj.2009.50.3.399 PMC2703764

[b23] Castilla C. *et al.* Immunohistochemical Expression of Hsp60 Correlates With Tumor Progression and Hormone Resistance in Prostate Cancer. Urology 76, 1017.e1–1017.e6 (2010). 20708221 10.1016/j.urology.2010.05.045

[b24] Hamrita B. *et al.* Identification of tumor antigens that elicit a humoral immune response in breast cancer patients’ sera by serological proteome analysis (SERPA). Clin. Chim. Acta 393, 95–102 (2008). 18424265 10.1016/j.cca.2008.03.017

[b25] Ghosh J. C., Dohi T., Kang B. H. & Altieri D. C. Hsp60 regulation of tumor cell apoptosis. J. Biol. Chem. 283, 5188–5194 (2008). 18086682 10.1074/jbc.M705904200

[b26] Sabharwal S. S. & Schumacker P. T. Mitochondrial ROS in cancer: initiators, amplifiers or an Achilles’ heel? Nat. Rev. Cancer 14, 709–721 (2014). 25342630 10.1038/nrc3803 PMC4657553

[b27] Krishan S., Richardson D. R. & Sahni S. Adenosine monophosphate-activated kinase and its key role in catabolism: structure, regulation, biological activity, and pharmacological activation. Mol. Pharmacol. 87, 363–377 (2015). 25422142 10.1124/mol.114.095810

[b28] Roy L. D. *et al.* MUC1 enhances invasiveness of pancreatic cancer cells by inducing epithelial to mesenchymal transition. Oncogene 30, 1449–1459 (2011). 21102519 10.1038/onc.2010.526 PMC3063863

[b29] Xu L., Chow K. K. H., Lim M. & Li G. Current Vaccine Trials in Glioblastoma: A Review. J. Immunol Res. (2014). 10.1155/2014/796856 PMC3996322 24804271

[b30] Stupp R. *et al.* Radiotherapy plus concomitant and adjuvant temozolomide for glioblastoma. New Engl. J. Med. 352, 987–996 (2005). 15758009 10.1056/NEJMoa043330

[b31] Stupp R. *et al.* Effects of radiotherapy with concomitant and adjuvant temozolomide versus radiotherapy alone on survival in glioblastoma in a randomised phase III study: 5-year analysis of the EORTC-NCIC trial. Lancet Oncol. 10, 459–466 (2009). 19269895 10.1016/S1470-2045(09)70025-7

[b32] Ghosh J. C., Siegelin M. D., Dohi T. & Altieri D. C. Heat shock protein 60 regulation of the mitochondrial permeability transition pore in tumor cells. Cancer Res. 70, 8988–8993 (2010). 20978188 10.1158/0008-5472.CAN-10-2225 PMC2982903

[b33] Carroll J. *et al.* Bovine complex I is a complex of 45 different subunits. J. Biol. Chem. 281, 32724–32727 (2006). 16950771 10.1074/jbc.M607135200

[b34] Sabharwal S. S. & Schumacker P. T. Mitochondrial ROS in cancer: initiators, amplifiers or an Achilles’ heel? Nat. Rev. Cancer 14, 709–721 (2014). 25342630 10.1038/nrc3803 PMC4657553

[b35] Rigoulet M., Yoboue E. D. & Devin A. Mitochondrial ROS Generation and Its Regulation: Mechanisms Involved in H_2_O_2_ Signaling. Antioxid. Redox Sign. 14, 459–468 (2011). 10.1089/ars.2010.3363 20649461

[b36] Sharma L. K. *et al.* Mitochondrial respiratory complex I dysfunction promotes tumorigenesis through ROS alteration and AKT activation. Hum. Mol. Genet. 20, 4605–4616 (2011). 21890492 10.1093/hmg/ddr395 PMC3209831

[b37] Hardie D. G. AMP-activated/SNF1 protein kinases: conserved guardians of cellular energy. Nat. Rev. Mol. Cell Biol. 8, 774–785 (2007). 17712357 10.1038/nrm2249

[b38] Steinberg G. R. & Kemp B. E. AMPK in Health and Disease. Physiol. Rev. 89, 1025–1078 (2009). 19584320 10.1152/physrev.00011.2008

[b39] Srivastava R. A. *et al.* AMP-activated protein kinase: an emerging drug target to regulate imbalances in lipid and carbohydrate metabolism to treat cardio-metabolic diseases. J. Lipid Res. 53, 2490–2514 (2012). 22798688 10.1194/jlr.R025882 PMC3494254

[b40] Ruderman N. B., Carling D., Prentki M. & Cacicedo J. M. AMPK, insulin resistance, and the metabolic syndrome. J. Clin. Invest. 123, 2764–2772 (2013). 23863634 10.1172/JCI67227 PMC3696539

[b41] Shaw R. J. LKB1 and AMP-activated protein kinase control of mTOR signalling and growth. Acta Physiol. (Oxf) 196, 65–80 (2009). 19245654 10.1111/j.1748-1716.2009.01972.x PMC2760308

[b42] Inoki K., Li Y., Xu T. & Guan K. L. Rheb GTPase is a direct target of TSC2 GAP activity and regulates mTOR signaling. Gene Dev. 17, 1829–1834 (2003). 12869586 10.1101/gad.1110003 PMC196227

[b43] Dibble C. C. & Manning B. D. Signal integration by mTORC1 coordinates nutrient input with biosynthetic output. Nat. Cell Biol. 15, 555–564 (2013). 23728461 10.1038/ncb2763 PMC3743096

[b44] Gwinn D. M. *et al.* AMPK phosphorylation of raptor mediates a metabolic checkpoint. Mol. Cell 30, 214–226 (2008). 18439900 10.1016/j.molcel.2008.03.003 PMC2674027

[b45] Wullschleger S., Loewith R. & Hall M. N. TOR signaling in growth and metabolism. Cell 124, 471–484 (2006). 16469695 10.1016/j.cell.2006.01.016

[b46] Holz M. K., Ballif B. A., Gygi S. P. & Blenis J. mTOR and S6K1 mediate assembly of the translation preinitiation complex through dynamic protein interchange and ordered phosphorylation events. Cell 123, 569–580 (2005). 16286006 10.1016/j.cell.2005.10.024

[b47] Haghighat A., Mader S., Pause A. & Sonenberg N. Repression of Cap-Dependent Translation by 4E-Binding Protein 1:Competition with P220 for Binding to Eukaryotic Initiation Factor-4E. EMBO J. 14, 5701–5709 (1995). 8521827 10.1002/j.1460-2075.1995.tb00257.x PMC394685

[b48] Gingras A. C. *et al.* Regulation of 4E-BP1 phosphorylation: a novel two-step mechanism. Gene Dev. 13, 1422–1437 (1999). 10364159 10.1101/gad.13.11.1422 PMC316780

[b49] Hardie D. G. & Pan D. A. Regulation of fatty acid synthesis and oxidation by the AMP-activated protein kinase. Biochem. Soc. Trans. 30, 1064–1070 (2002). 12440973 10.1042/bst0301064

[b50] Li Y. *et al.* AMPK phosphorylates and inhibits SREBP activity to attenuate hepatic steatosis and atherosclerosis in diet-induced insulin-resistant mice. Cell Metab. 13, 376–388 (2011). 21459323 10.1016/j.cmet.2011.03.009 PMC3086578

[b51] Tiedemann R. E. *et al.* Identification of molecular vulnerabilities in human multiple myeloma cells by RNA interference lethality screening of the druggable genome. Cancer Res. 72, 757–768 (2012). 22147262 10.1158/0008-5472.CAN-11-2781 PMC3622723

[b52] Gu L. *et al.* Functional Characterization of Sirtuin-like Protein in Mycobacterium smegmatis. J. Proteome Res. 14, 4441–4449 (2015). 26375486 10.1021/acs.jproteome.5b00359

[b53] Bligh E. G. & Dyer W. J. A rapid method of total lipid extraction and purification. Can. J. Biochem. Physiol. 37, 911–917 (1959). 13671378 10.1139/o59-099

