# Supplementary material for: Down-regulation of HSP60 Suppresses the Proliferation of Glioblastoma Cells via the ROS/AMPK/mTOR Pathway
Source: Sci Rep. 2016 Jun 21;6:28388. doi: 10.1038/srep28388 (PMC4914999; doi:10.1038/srep28388)
Supplement: Supplementary Information [file srep28388-s2.doc]

Down-regulation of HSP60 Suppresses the Proliferation of Glioblastoma Cells via the ROS/AMPK/mTOR Pathway

Haiping Tang^1^, Jin Li^1^, Xiaohui Liu^1^, Guihuai Wang^2^, Minkui Luo^3^, Haiteng Deng^1*^


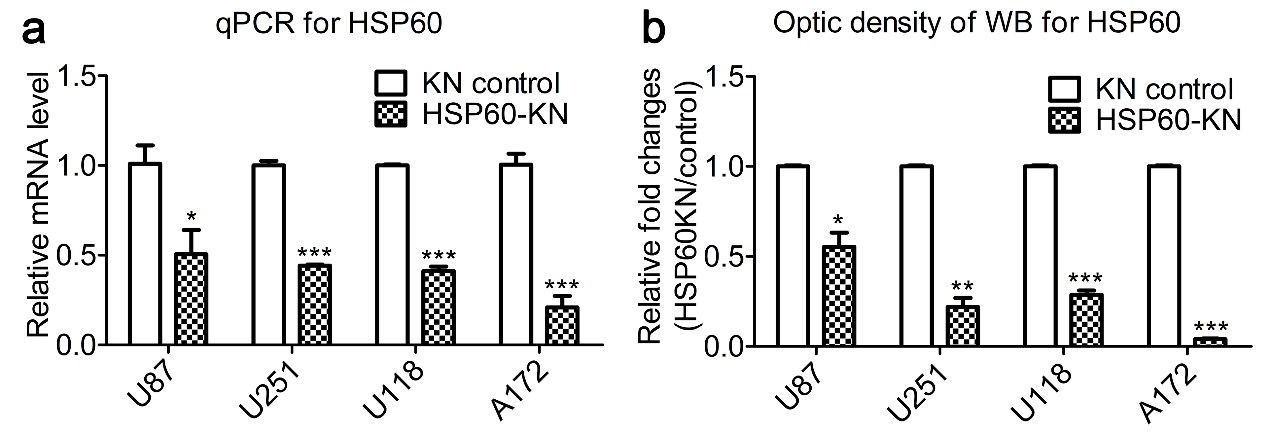


Supplementary Figure S1.The results of qPCR and optic density analysis of western blotting images of HSP60 in HSP60-KN cells. Data were analyzed using student’s t test. *p<0.05, **p<0.01 and *** p< 0.001. *p < 0.05 is considered statistically significant. Error bars represent ±SEM.


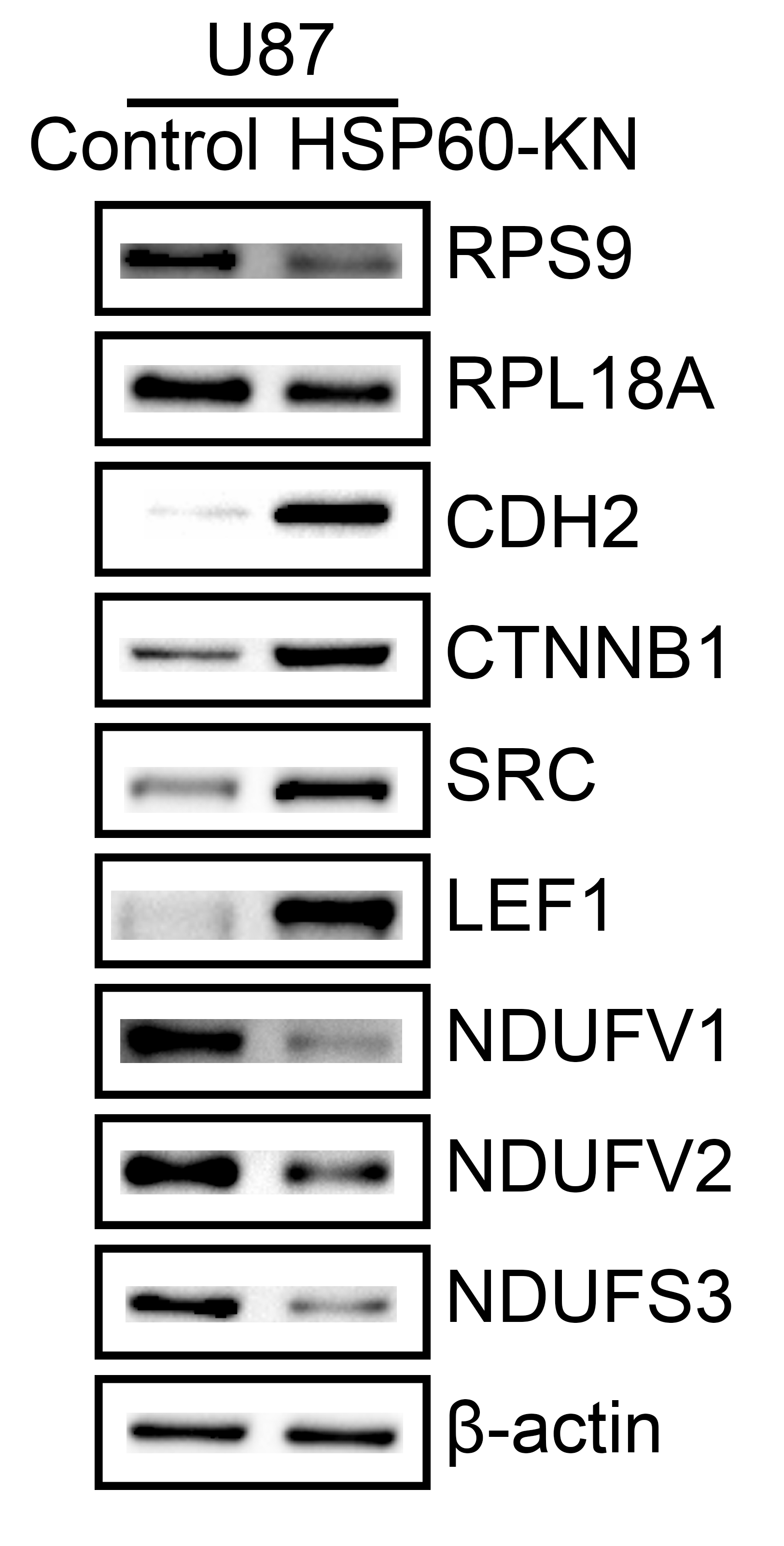
Supplementary Figure S2. Western blotting images of RPS9, RPL18A, CDH2, CTNNB1, SRC, LEF1, NDUFV1, NDUFV2 and NDUFS3 in the control and HSP60-KN-U87 cells.


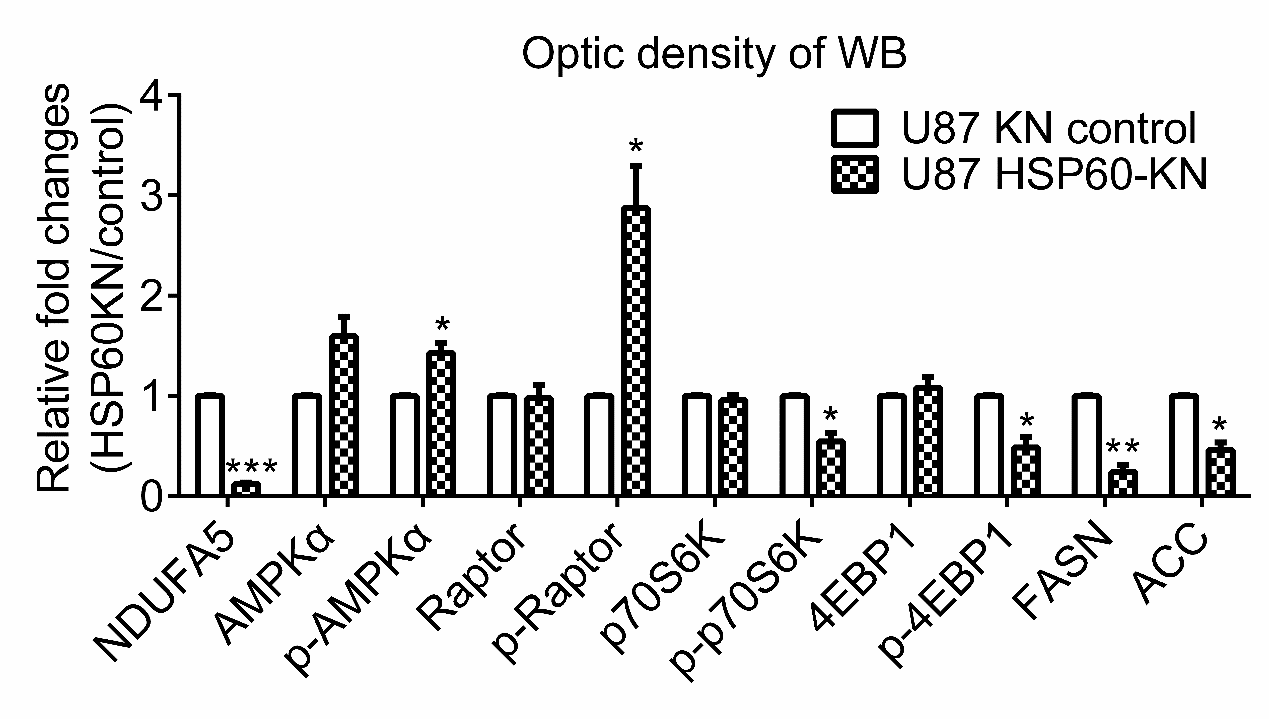


Supplementary Figure S3. The optic density analysis of western blotting images of NDUFA5, AMPKα, p-AMPKα-T172, Raptor, p-Raptor-S792, p70S6K, p-p70S6K-T389, 4EBP1, p-4EBP1-T37/46, FASN and ACC in HSP60-KN-U87 cells. Data were analyzed using student’s t test. *p<0.05, **p<0.01 and *** p< 0.001. *p < 0.05 is considered statistically significant. Error bars represent ±SEM.


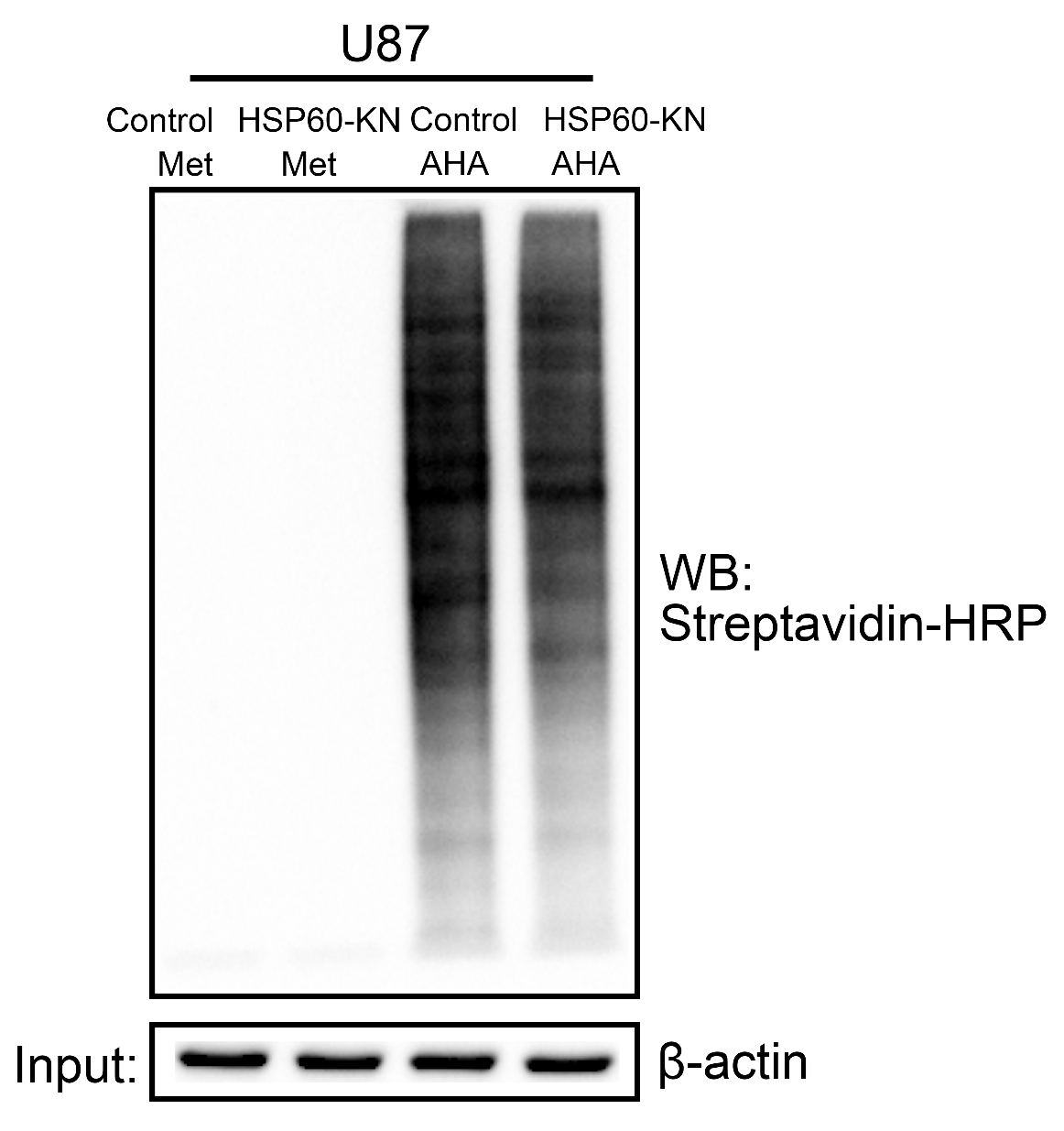


Supplementary Figure S4. Western blot analysis of AHA labeled proteins.


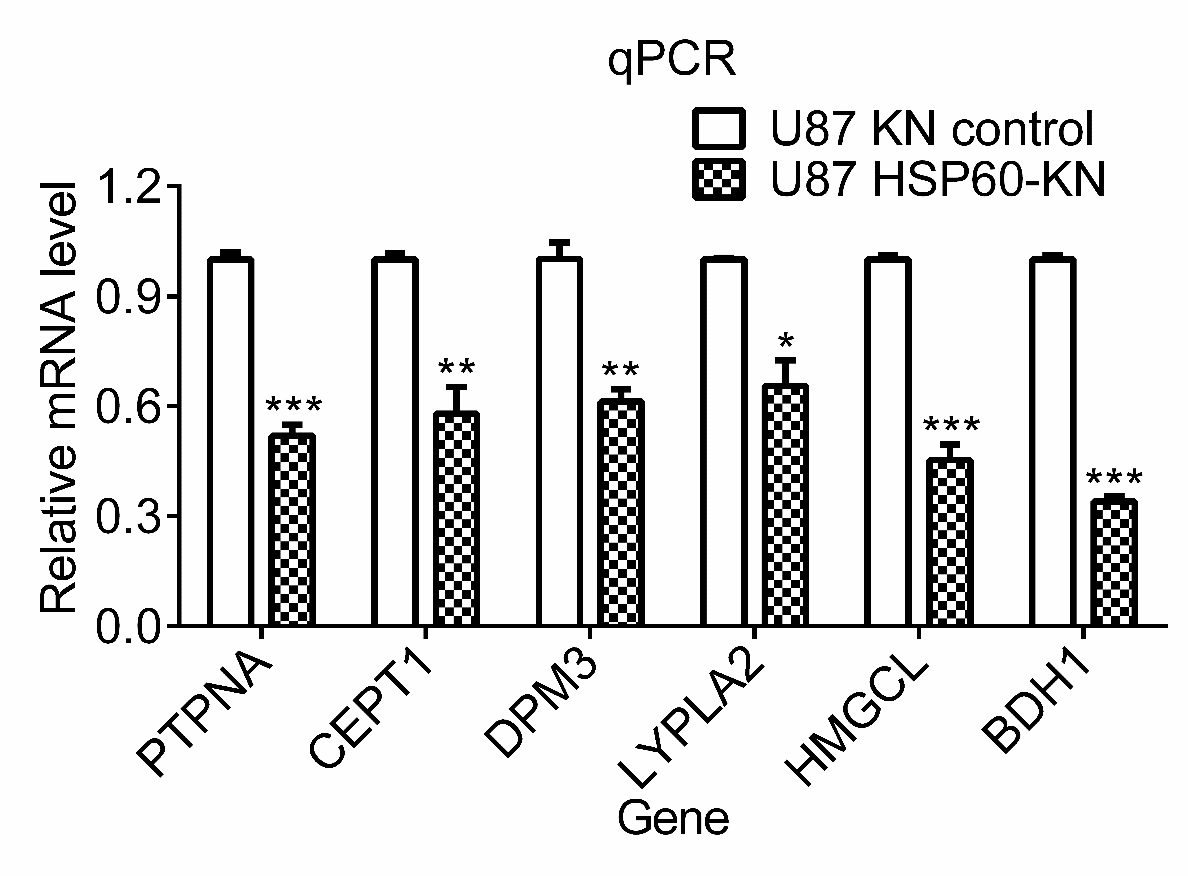


Supplementary Figure S5. qPCR validation of proteins related to lipid metabolism in the control and HSP60-KN-U87 cells. Data were analyzed using student’s t test. *p<0.05, **p<0.01 and *** p< 0.001. *p < 0.05 is considered statistically significant. Error bars represent ±SEM.


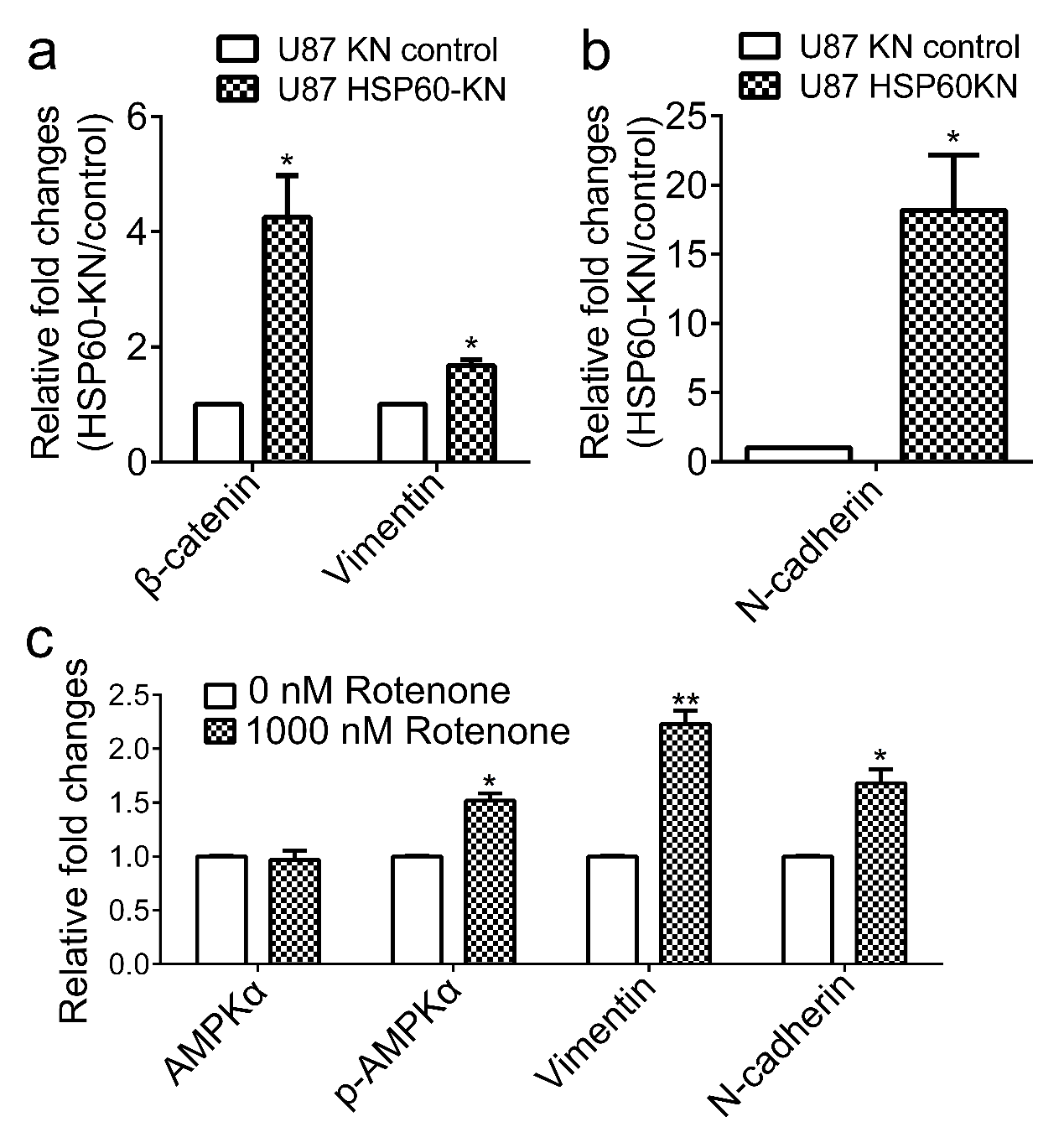


Supplementary Figure S6. The optic density analysis of western blotting images of EMT markers and AMPK in Figure 6. Data were analyzed using student’s t test. *p<0.05, **p<0.01 and *** p< 0.001. *p < 0.05 is considered statistically significant. Error bars represent ±SEM.


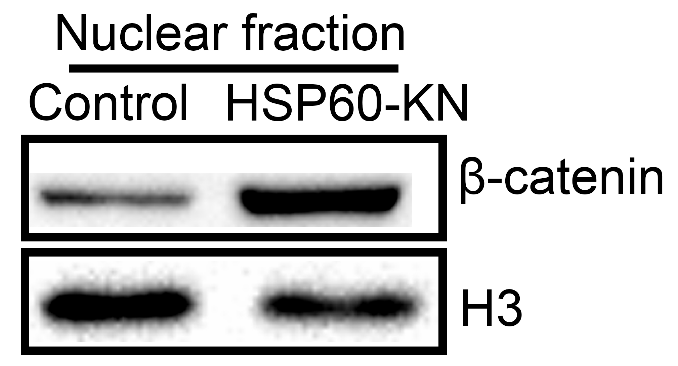


Supplementary Figure S7. Western blotting images of β-catenin in nuclear fractions.
